# Use the Right Looking Glass When You Do Caliper-verified Kinematically Aligned TKA!

**DOI:** 10.1016/j.artd.2022.04.003

**Published:** 2022-05-10

**Authors:** Stephen M. Howell

**Affiliations:** Department of Biomedical Engineering, University of California, Davis, Adventist Health/Lodi Memorial Hospital, Sacramento CA, USA

“Through the Looking-Glass” refers to the Lewis Carroll novel (ie, the sequel to Alice in Wonderland) where Alice crosses over into a “bizarre universe” when she enters the flipped world on the other side of a mirror. The phrase implies unpredictable and strange happenings, a poignant metaphor when the mechanical alignment (MA) total knee arthroplasty (TKA) surgeon confronts kinematic alignment (KA) for the first time!

Interest in caliper-verified KA TKA is justifiably growing. 11 of 13 randomized trials, case-matched studies, and case series of bilateral TKA with a KA TKA and MA TKA from centers worldwide, including a 2022 publication from the Hospital for Special Surgery [1], show better clinical outcome scores, knee range of motion, alignment, fewer ligament releases, or better knee kinematics than MA TKA. Equally important are the 2021 Australian and New Zealand registry results, and a case series study showed implant survival is comparable or better than MA at 7 to 10 years, respectively [2,3].

The concept of caliper-verified KA is simple. The KA surgeon adopts the dentist's 3-D approach of fitting a crown on the worn tooth by focusing on surface relationships inside the knee and not on distant landmarks (ie, the femoral head and ankle centers) or a preoperative radiographic image.

For the femur, the surgeon sets the femoral component coincident to the patient's distal and posterior prearthritic joint lines without releasing ligaments, including the PCL. First, the surgeon computes the femoral resection target to restore the joint lines by subtracting 1 mm for the saw blade's kerf and 2 mm for complete cartilage loss from the thickness of the condyle of the femoral component. Next, intraoperative caliper measurements determine whether the thickness of these femoral bone resections is within the ±0.5 mm of the KA target. Performing the caliper measurements identifies unintended target deviations, enabling a correction before femoral component implantation. A 2022 report analyzing over 1000 femoral resections performed using manual instruments by less-experienced and experienced surgeons showed comparable and greater accuracy than the femoral resections performed by robotics [4].

For the tibia, the surgeon uses the gap-balance technique to fine-tune the tibial resection until a check with a spacer block and trial components verifies a rectangular extension space with negligible varus-valgus laxity. Simultaneously, the surgeon cuts the slope to match the patient's medial prearthritic posterior slope when retaining the PCL. These steps, by default, restore medial and lateral tibial compartment forces and laxities close to those of the native knee. The postoperative radiographic expectation is that angular component alignment closely matches the contralateral knee joint.

Surgeons transitioning from MA to caliper-verified KA need to appreciate the unresolvable conflict between the 2 alignment philosophies. As F. Scott Fitzgerald wrote, “The test of a first-rate intelligence is the ability to follow 2 opposing ideas simultaneously, and still retain the ability to function.” Those that blend the philosophies experience frustration that compromises the patient's result. Hence, it is best to follow all the KA principles and use the correct “looking glass” to assess the intraoperative and radiographic component positions.

When an MA-trained surgeon operates in the “bizarre universe” of KA, they slowly learn to “see” the knee differently. No longer are components set to the MA targets of the femoral head and ankle, the transepicondylar axis and Whiteside's line, and a fixed posterior slope. The need for releasing ligaments disappears as contraction and stretching are strikingly rare, even in the most deformed knees. Correction of severe varus and valgus deformities becomes more straightforward, and flexion contractures readily resolve by releasing the posterior capsule and, when needed, passively manipulating the knee into extension.

They gain confidence from using KA when treating the contralateral knee of patients with a MA TKA, as the subsequent KA TKA’s recovery is more often faster and less painful with shorter narcotic use. Furthermore, the KA TKA's Forgotten Joint Score is higher and comparable to that of a total hip replacement [5,6]. These encouraging KA results and a surgery freed from the morbidity associated with ligament release enable the transition from an overnight hospital stay to same-day discharge to home, which we have done since July 2020 in nearly all patients. In addition, KA patients realize that forgoing formal physical therapy and performing self-administering motion exercises provide a less painful and faster recovery.

MA surgeons always ask whether there is an “acceptable” range for the components' varus, valgus, and posterior slope after KA. This question is irrelevant to the KA surgeon because restoring the patient's prearthritic joint lines means no components are set in varus or valgus or to a fixed value. Instead, imagine the MA surgeon uses the KA looking glass to examine their component positions. Based on Hirschmann's phenotype studies, they will realize that MA's “one-approach fits all” cuts 84% of the distal femurs in varus and 70% of the proximal tibias in valgus relative to the patient's prearthritic joint lines [5,6]. The more frequent femoral varus cut than a tibial valgus cut causes medial overload and a high knee adduction moment in 14% of MA TKA, which explains why MA causes varus tibial component failure and KA does not. In addition, MA changes the Q-angle, whereas KA does not. In those patients with constitutional varus, which makes up 70% of the population, the increase in the q-angle could compromise patellofemoral tracking. Therefore, it is not unexpected that the risk of patellofemoral complications is comparable after KA and MA, even though KA sets the femoral component more internally rotated than MA.

Surgeons adopting KA go through a learning curve, and starting with patients with simple varus deformities eases concerns. When the surgeon is uncomfortable in setting components coincident with the patient's prearthritic joint lines, it is better for them and the patients to use MA than KA. Restricting correction can result in knee instability, high tibial compartment forces, and a limb alignment unnatural to the patient.

In conclusion, 2 implant designs are Food and Drug Administration and CE (conformité européenne -https://europa.eu/youreurope/business/product-requirements/labels-markings/ce-marking/index_en.htm) approved for use with caliper-verified KA, and this fosters a spirited competition between the KA and MA techniques. Use the KA looking glass when assessing the intraoperative and radiographic component position, and unpredictable and strange happenings will disappear!

## Conflicts of interest

The authors declare the following financial interests/personal relationships which may be considered as potential competing interests: The author is a consultant for and receives royalties from Medacta. The author also receives royalties from Elsevier.

For full disclosure statements refer to https://doi.org/10.1016/j.artd.2022.04.003.

## Appendix A Supplementary Data

Conflict of Interest Statement for Howell
