# Supplementary material for: Use the Right Looking Glass When You Do Caliper-verified Kinematically Aligned TKA!
Source: Arthroplast Today. 2022 May 10;15:139–40. doi: 10.1016/j.artd.2022.04.003 (PMC9108522; doi:10.1016/j.artd.2022.04.003)
Supplement: Conflict of Interest Statement for Howell [file mmc1.pdf]

CONFLICT OF INTEREST STATEMENT

American Association of Hip and Knee Surgeons

(Adopted from the American Academy of Orthopaedic Surgeons disclosure statement)

The following form **must be filled out completely and submitted by each author (example, 6 authors, 6 forms). All items require a response. If there is no relevant disclosure for a given item, enter "*None.*"**

Use the Right Looking-Glass When You Do Caliper Verified Kinematically Aligned TKA!

Manuscript Title

1. Royalties from a company or supplier (The following conflicts were disclosed)

Medacta

2. Speakers bureau/paid presentations for a company or supplier (The following conflicts were disclosed)

Medacta

3A. Paid employee for a company or supplier (The following conflicts were disclosed)

None

3B. Paid consultant for a company or supplier (The following conflicts were disclosed)

Medacta

3C. Unpaid consultants for a company or supplier (The following conflicts were disclosed)

None

4. Stock or stock options in a company or supplier (The following conflicts were disclosed)

None

5. Research support from a company or supplier as a Principal Investigator (The following conflicts were disclosed)

Medacta

6. Other financial or material support from a company or supplier (The following conflicts were disclosed)

None

7. Royalties, financial or material support from publishers (The following conflicts were disclosed)

Elsevier

8. Medical/Orthopaedic publications editorial/governing board (The following conflicts were disclosed)

None

9. Board member/committee appointments for a society (The following conflicts were disclosed)

None

Each author must sign AND print or type his/her name, date and submit a separate form

In addition, one BLINDED Conflict of Interest form (no author names used) should be submitted per manuscript with all author disclosures.

Stephen M Howell, MD | *Stephen M Howell MD* | 3/29/22

Author Name (Print or Type) | Author Signature | Date
